# Key to Chinese species of stenocranine planthoppers (Hemiptera, Fulgoromorpha, Delphacidae), with descriptions of two new species

**DOI:** 10.3897/zookeys.1279.177978

**Published:** 2026-05-18

**Authors:** Min Tang, Nian Gong, Zi-Zhong Li, Lin Yang, Sha-Sha Lv, Jian-Kun Long, Zhi-Min Chang, Xiao Feng, Mao-Heng Du, Xiang-Sheng Chen

**Affiliations:** 1 The Provincial Key Laboratory of Agricultural Biosecurity of Guizhou, Guizhou University, Guiyang, Guizhou, 550025, China Institute of Entomology, Guizhou University Guiyang China; 2 Institute of Entomology, Guizhou University, Guiyang, Guizhou, 550025, China The Provincial Key Laboratory of Agricultural Biosecurity of Guizhou, Guizhou University Guiyang China; 3 The Provincial Special Key Laboratory for Development and Utilization of Insect Resources of Guizhou, Guizhou University, Guiyang, Guizhou, 550025, China The Provincial Special Key Laboratory for Development and Utilization of Insect Resources of Guizhou, Guizhou University Guiyang China; 4 Guizhou Provincial Engineering Research Center of Medical Resourceful Healthcare Products, Guiyang Healthcare Vocational University, Guiyang, Guizhou, 550081, China Guizhou Provincial Engineering Research Center of Medical Resourceful Healthcare Products, Guiyang Healthcare Vocational University Guiyang China; 5 The Administrations of Fodingshan National Nature Reserve, Tongren, Guizhou, 554300, China The Administrations of Fodingshan National Nature Reserve Tongren China

**Keywords:** Fulgoroidea, identification key, morphology, phytophagous, planthopper, Stenocraninae, taxonomy

## Abstract

Two new species of the genus *Stenocranus* Fieber, 1866 and *Ceratocranus* Fujinuma & Hayashi, 2025, *S. fodingshanensis* Tang, Li & Chen, **sp. nov**. and *C. huaxiensis* Tang, Li & Chen, **sp. nov**., are described and illustrated from China (Guizhou). A key to all known Chinese *Stenocranus* and *Ceratocranus* species, and ecological photos of the two new species, are provided.

## Introduction

The planthoppers of the subfamily Stenocraninae Wagner, 1963 (Hemiptera, Fulgoromorpha, Delphacidae) represent a relatively small group primarily distributed in the Oriental and Palaearctic regions (Wagner 1963; Ding 2006). Currently, Stenocraninae includes 84 species in 11 genera, of which 30 species in four genera occur in China (Bartlett 2005, 2009; Bourgoin 2024; Fujinuma and Hayashi 2025). These phytophagous insects cause direct damage to crops by feeding on phloem sap and indirectly by transmitting plant viral diseases (Bartlett et al. 2018). The majority of stenocranine delphacids have been reported to feed on Poaceae or Cyperaceae that are important cash crops (Bartlett and Wheeler 2007). For example, *Stenocranus yuanmaonus* Kuoh, 1980 and *S. matsumurai* Metcalf, 1943 are important pests of *Phragmites australis* (Cav.) Trin. ex Steud, 1841 (Poaceae, Arundinoideae) in Baiyangdian (Hebei, China), and have a significant economic impact on the production and utilization of these reeds (Ren 1988; Li et al. 2006).

The genus *Stenocranus* Fieber, 1866 was established for four species, with *S. minutus* (Fabricius, 1787) as the type species (Fieber 1866). Subsequently, many scholars have conducted research on *Stenocranus*. For instance, Crawford (1914) and Metcalf (1923, 1954) described six new species in the Americas; Muir (1916, 1917, 1923, 1929) described seven new species; Matsumura (1935a, 1935b) and Ishihara (1952a, 1952b) described 16 new species in Japan; Beamer (1946) described 13 species in North America, including five new species; Kuoh et al. (1980), Ding and Kuoh (1981), Ding and Zhang (1994), Chen and Liang (2005), and Ding (2006) described a total of 20 new species from China; and Hamilton (2006) reported ten species of *Stenocranus* from Canada. Furthermore, Fujinuma and Hayashi (2025) revised *Stenocranus* in Japan and established a new genus *Ceratocranus*, describing 12 species, including 2 new species. To date, 61 species have been recorded in *Stenocranus*, which is widely distributed in the Oriental and Palaearctic regions, with 25 species in China (Kuoh et al. 1980, 1984; Ding and Kuoh 1981; Yang 1989; Ding and Zhang 1994; Wang and Ding 1996; Chen and Liang 2005; Ding 2006; Qin et al. 2010; Bourgoin 2024).

While sorting and identifying recently collected specimens, two new species of *Stenocranus* and *Ceratocranus* were discovered: *S. fodingshanensis* Tang, Li & Chen, sp. nov. and *C. huaxiensis* Tang, Li & Chen, sp. nov., both from Guizhou Province, China. In the present paper, the new species are described and illustrated, a key to the known 28 Chinese species of *Stenocranus* and *Ceratocranus* is provided, along with photographs of the collecting sites of the two species.

## Material and methods

The external morphology terminologies are as follows: male genitalia follows Yang (1989), Ding (2006) and Zhou et al. (2019), female genitalia follows Bourgoin (1993), and wing venation follows Bourgoin et al. (2015). Dry male specimens were used for the descriptions and illustrations. Body measurements are from the apex of the vertex to the apex of the forewing. All measurements are in millimeters (mm). External morphology was observed under a stereoscopic microscope and characters were measured with an ocular micrometer. Color pictures for the adult habitus were obtained using the KEYENCE VHX-1000 system. The genital segments of the examined specimens were macerated in 10% NaOH and drawn from preparations in glycerin jelly using a Leica MZ 12.5 stereo microscope. Illustrations were scanned with a CanoScan LiDE 200 and imported into Adobe Photoshop CC and Adobe Illustrator 2020 for labeling and plate composition. The dissected male genitalia are preserved in glycerin jelly in small plastic tubes pinned together with the specimens.

The type specimens of the new species are deposited in the Institute of Entomology, Guizhou University, Guiyang, China (IEGU).

## Taxonomy

### Stenocranus

Taxon classification

Animalia

HemipteraDelphacidae

Fieber, 1866

05E5E963-A6F9-5EB5-B25A-9FBA234D79A9

Stenocranus Fieber, 1866: 519; Matsumura 1935a: 125; Matsumura 1935b: 71; Matsumura and Ishihara 1945: 68; Ishihara 1949: 23; Kuoh et al. 1984: 88; Yang 1989: 12; Ding and Zhang 1994: 23; Wang and Ding 1996: 43; Chen and Liang 2005: 124; Ding 2006: 81; Qin et al. 2010: 925; Fujinuma and Hayashi 2025: 302.

#### Type species.

*Fulgora minutus* Fabricius, 1787, original designation.

#### Diagnosis.

Head including eyes narrower than pronotum. Vertex oblong, longer in middle line than wide at base, submedian carinae uniting at apex, Y-shaped carina distinct or obscure. Frons longer in middle line than wide at widest part, more than 2:1, widest at middle, median carina simple. Antennae short, reaching frontoclypeal suture, pedicel about 3X or longer than scape. Pronotum shorter than vertex, lateral carinae slightly curved, attaining hind margin. Metatibiae with two lateral spines. Hind legs with spinulation: tibial apex with 5 (2+3) spinules, basitarsus with 7 (2+5) spinules, second tarsomere with 4 (rarely 5) spinules. Post-tibial spur dilated, flattened, numerous small teeth with rectangular platelike base on inner margin, shorter than basitarsus, with about 12–26 teeth on posterior margin.

***Male genitalia***: Anal segment of male ring-like, lateroapical angles each produced into process or not. Pygofer without medioventral process. Aedeagus with phallobase, distinct, phallobasal process slender, curved, phallus slender, rod-like. Suspensorium various. Diaphragm rather broad. Genital styles moderately long (Yang 1989).

***Female genitalia***: Pygofer nearly as long as gonoplacs in ventral view, usually mostly covered with gonoplacs. Gonocoxae VIII broad in ventral view, produced medially at base, narrowed to apices. Gonapophyses VIII slender in ventral view, nearly smooth to strongly serrated on lateroapical parts. Gonapophyses IX broad in dorsal view, dorsal margin with variously shaped teeth in apical 1/3 to 2/3. Gonoplacs usually very broad (rarely slender) in ventral view, semicircular (Fujinuma and Hayashi 2025).

#### Distribution.

Australian, Oriental, Palearctic and Nearctic regions.

##### Key to Chinese species (males) of the genus *Stenocranus* (revised from Ding 2006)

**Table:** 

1	Frons between carinae light-colored or with two faint linear dark colored stripes at base	**2**
–	Frons between carinae entirely black, or with black stripes extending from base to apex (Fig. 1B, F)	**5**
2	Anal segment with spinose processes (Figs 2I, 2J, 3F, 3G)	**3**
–	Anal segment without spinose processes (Yang 1989: fig. 1C, D)	***S. niisimai* Matsumura, 1935**
3	Phallobase branched at apex (Kuoh et al. 1980: fig. 1f)	***S. hongtiaus* Kuoh, 1980**
–	Phallobase unbranched at apex	**4**
4	Anal segment with two pairs of spinose processes (Kuoh et al. 1980: fig. 2f)	***S. yuanmaonus* Kuoh, 1980**
–	Anal segment with three pairs of spinose processes (Ding and Zhang 1994: fig. 20H)	***S. spinosus* Ding, 1994**
5	Forewings testaceous or posterior half of membrane infuscated (Fig. 1H)	**6**
–	Forewings mostly fuscous or membrane fuscous or membrane near hind margin with a fuscous longitudinal stripe (Fig. 1D)	**11**
6	Forewings with veins concolorous, apical veins without dark spots	**7**
–	Forewings with veins darker than membrane, apical veins with dark spots (Fig. 1D, H)	**9**
7	Vertex, pro- and mesonotum with a light-colored median longitudinal stripe (Ding and Kuoh 1981: fig. 25)	***S. testaceus* Ding, 1981**
–	Vertex, pro- and mesonotum without a light-colored median longitudinal stripe	**8**
8	Anal segment collar-like, uniting at basal lateroapical angles; inner margin of genital styles with an angular process (Ding and Kuoh 1981: figs 22, 23)	***S. castaneus* Ding, 1981**
–	Anal segment arch-shaped, not uniting at basal lateroapical angles; inner margin of genital styles with an arcuate process at middle part (Ding 2006: fig. 41E, F)	***S. tonghuaensis* Ding, 2006**
9	Anal segment with a pair of spinose processes (Qin et al. 2010: figs 11, 13)	***S. pacificus* Kirkaldy, 1907**
–	Anal segment without spinose processes	**10**
10	Genital styles forming an oval shape, in ventral view	***S. fallax* Matsumura, 1935**
–	Genital styles not as above	***S. takasagonis* Matsumura, 1935**
11	Forewings mostly fuscous or membrane fuscous	**12**
–	Forewings with membrane near hind margins with a fuscous longitudinal stripe (Fig. 1C, D)	**14**
12	Forewings mostly fuscous, with a few hyaline markings	**13**
–	Forewings only membrane fuscous (Ding 2006: fig. 40K)	***S. cyperi* Ding, 2006**
13	Dorsum of vertex and thorax with light-colored median longitudinal stripe; anal segment of male without spinose processes (Ding 2006: fig. 39A, D, I)	***S. jiangpuensis* Ding, 2006**
–	Dorsum of vertex and thorax without light-colored median longitudinal stripe; anal segment with two pairs of spinose processes (Kuoh et al. 1980: fig. 5a, c)	***S. montanus* Huang & Ding, 1980**
14	Anal segment with spinose process (Figs 2I, 2J, 3F, 3G)	**15**
–	Anal segment without spinose process	**20**
15	Anal segment with two pairs of spinose processes (Ding 2006: fig. 37H)	***S. zalantunensis* Ding & Hu, 1994**
–	Anal segment of male with one pair of spinose processes	**16**
16	Anal segment with thick spinose processes; genital styles inner angle with slender process (Chen and Liang 2005: figs 6, 10, 11)	***S. anomalus* Chen & Liang, 2005**
–	Anal segment with short spinose processes; genital styles inner angle without slender process	**17**
17	Anal segment with spinose processes arising from apical lateral margins	**18**
–	Anal segment with spinose processes arising from medial lateral margins	**19**
18	Pygofer with an angular process on lateral and ventral margins (Ding and Kuoh 1981: fig. 11)	***S. linearis* Ding, 1981**
–	Pygofer without an angular process on lateral and ventral margins	***S. fuscovittatus* (Stål, 1858)**
19	Phallobase deeply concave in middle, with two branches that bend reversely (Ding and Kuoh 1981: fig. 31)	***S. longicapitis* Ding, 1981**
–	Phallobase with two branches that turn ventrad apically (Fig. 2F)	***S. fodingshanensis* Tang, Li & Chen, sp. nov**.
20	Black-brown stripes between carinae of frons narrow, at most half the width of the widest part between carinae	**21**
–	Black-brown stripes between carinae of frons wide, exceeding half the width of the widest part between carinae	**22**
21	Phallobase with three apical processes (Ding and Kuoh 1981: fig. 6)	***S. nigrocaudatus* Ding, 1981**
–	Phallobase with one apical process (Yang 1989: fig. 4H)	***S. planus* Yang, 1989**
22	Forewings with T-shaped markings on cross vein near hind margin (Ding 2006: fig. 31I)	***S. chenzhouensis* Ding, 1981**
–	Forewings without T-shaped markings on cross vein near hind margin	**23**
23	Lateral carinae of mesonotum with distinct fuscous stripes near lateral margins	**24**
–	Lateral carinae of mesonotum testaceous near lateral margins (Fig. 1E, G)	**25**
24	Phallobase with one curved apical process (Kuoh et al. 1980: fig. 3f)	***S. qiandainus* Kuoh, 1980**
–	Phallobase with two curved apical processes	***S. harimensis* Matsumura, 1935**
25	Phallobase tube-shaped, with two slender curved processes arising from the apical dorsal margin (Kuoh et al. 1980: fig. 4f)	***S. danjicus* Kuoh, 1980**
–	Phallobase not as above	***S. hopponis* Matsumura, 1935**

### Stenocranus fodingshanensis

Taxon classification

Animalia

HemipteraDelphacidae

Tang, Li & Chen sp. nov.

DF5302B9-36FE-5596-9E88-207A898444DC

https://zoobank.org/D831F4D8-EEE1-46AA-A688-45AA639240F9

**Figure 1.**
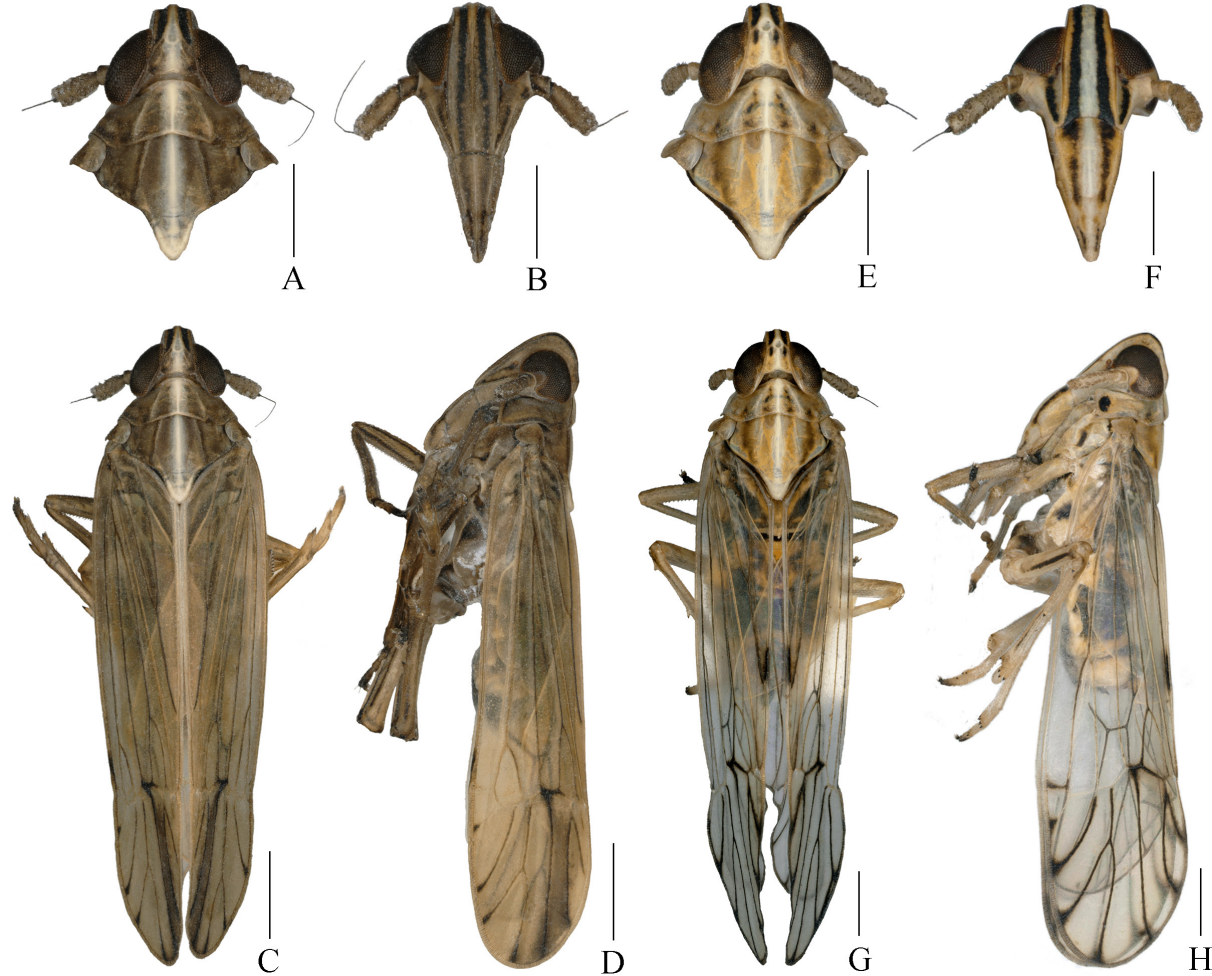
*Stenocranus fodingshanensis* Tang, Li & Chen, sp. nov. **A–D**. Male; **A**. Head and thorax, dorsal view; **B**. Frons, ventral view; **C**. Habitus, dorsal view; **D**. Habitus, lateral view. *Ceratocranus huaxiensis* Tang, Li & Chen, sp. nov. **E–H**. Male; **E**. Head and thorax, dorsal view; **F**. Frons, ventral view; **G**. Habitus, dorsal view; **H**. Habitus, lateral view. Scale bars: 0.5 mm**(A–H)**.

**Figure 2.**
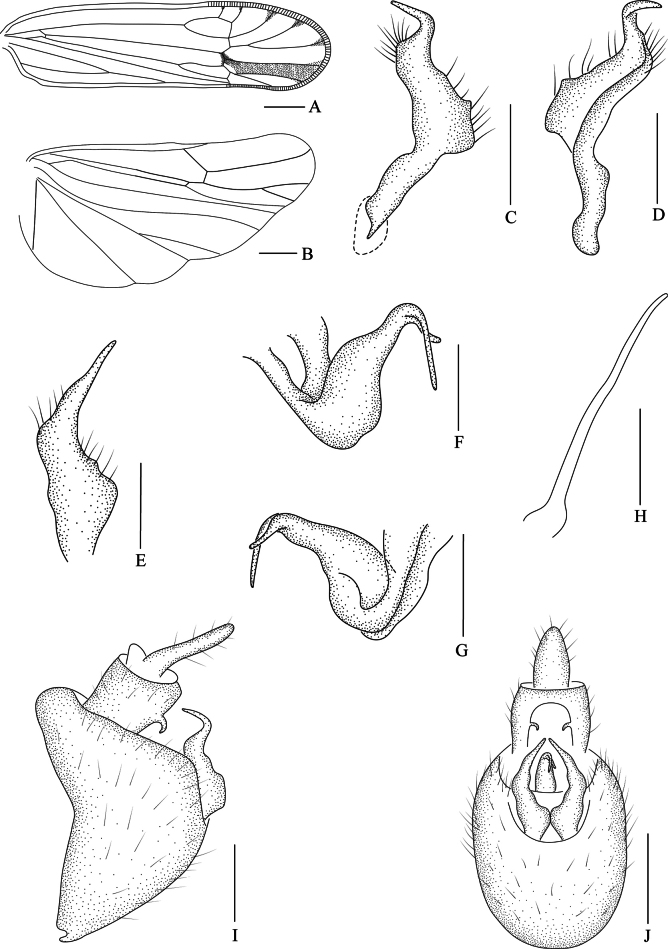
*Stenocranus fodingshanensis* Tang, Li & Chen, sp. nov., male. **A**. Forewing; **B**. Hindwing; **C**. Genital style, left lateral view; **D**. Genital style, right lateral view; **E**. Genital style, posterior view; **F**. Phallobase, left lateral view; **G**. Phallobase, right lateral view; **H**. Aedeagus, lateral view; **I**. Male genitalia, lateral view; **J**. Male genitalia, posterior view. Scale bars: 0.5 mm**(A**, **B)**; 0.2 mm**(C–J)**.

Figs 1A–D, 2, 4A–D, 5D–F

#### Type materials.

***Holotype***: China • ♂: Guizhou Province, Fodingshan National Nature Reserve; 27°33'N, 108°15'E; sweeping, 4 Aug. 2024; Min Tang leg.; IEGU. ***Paratypes***: China • 9♂♂11♀♀; same data as holotype; IEGU.

**Figure 3.**
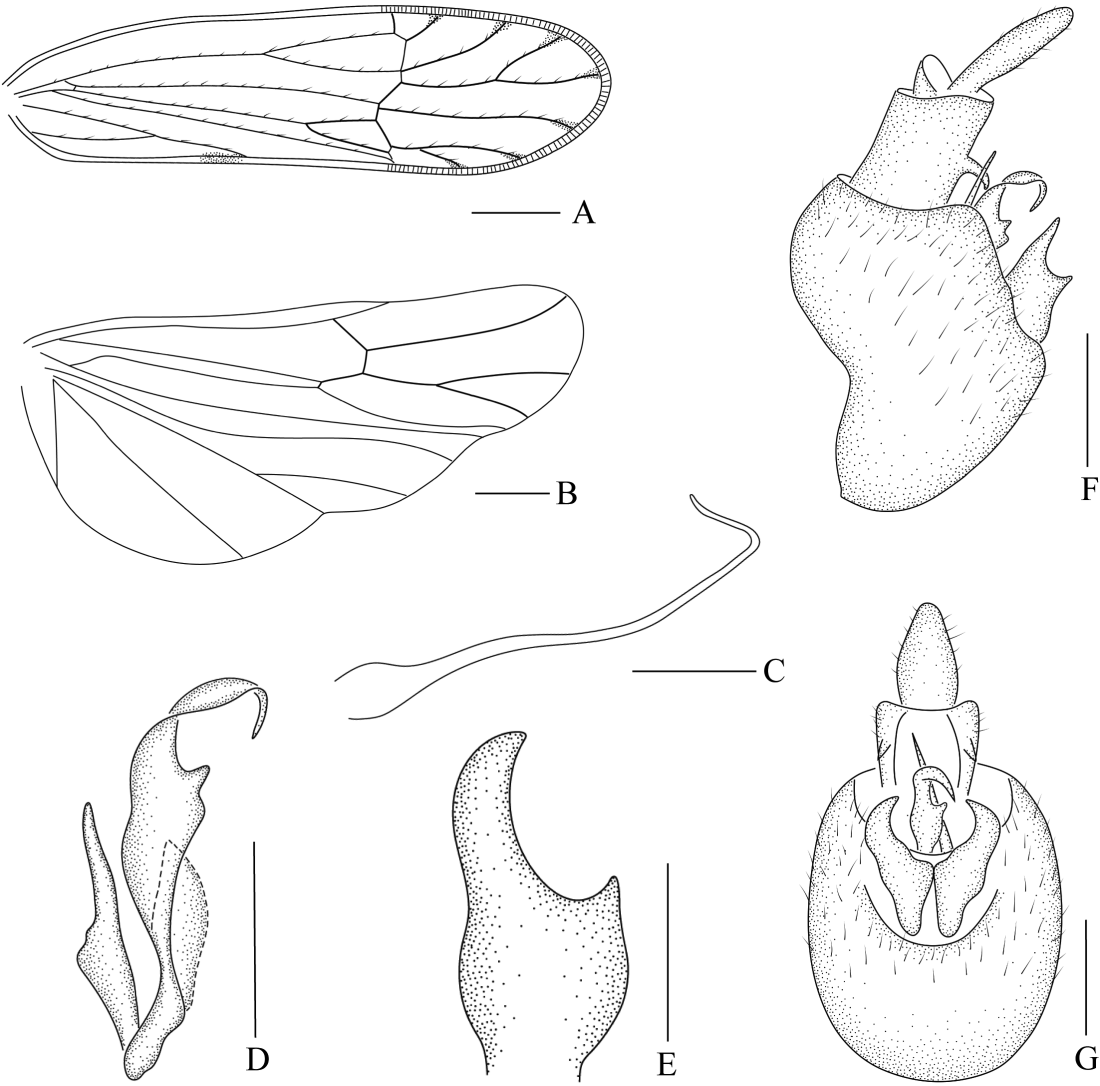
*Ceratocranus huaxiensis* Tang, Li & Chen, sp. nov., male. **A**. Forewing; **B**. Hindwing; **C**. Aedeagus, lateral view; **D**. Phallobase, lateral view; **E**. Genital style, posterior view; **F**. Male genitalia, lateral view; **G**. Male genitalia, posterior view. Scale bars: 0.5 mm**(A**, **B)**; 0.2 mm**(C–G)**.

**Figure 4.**
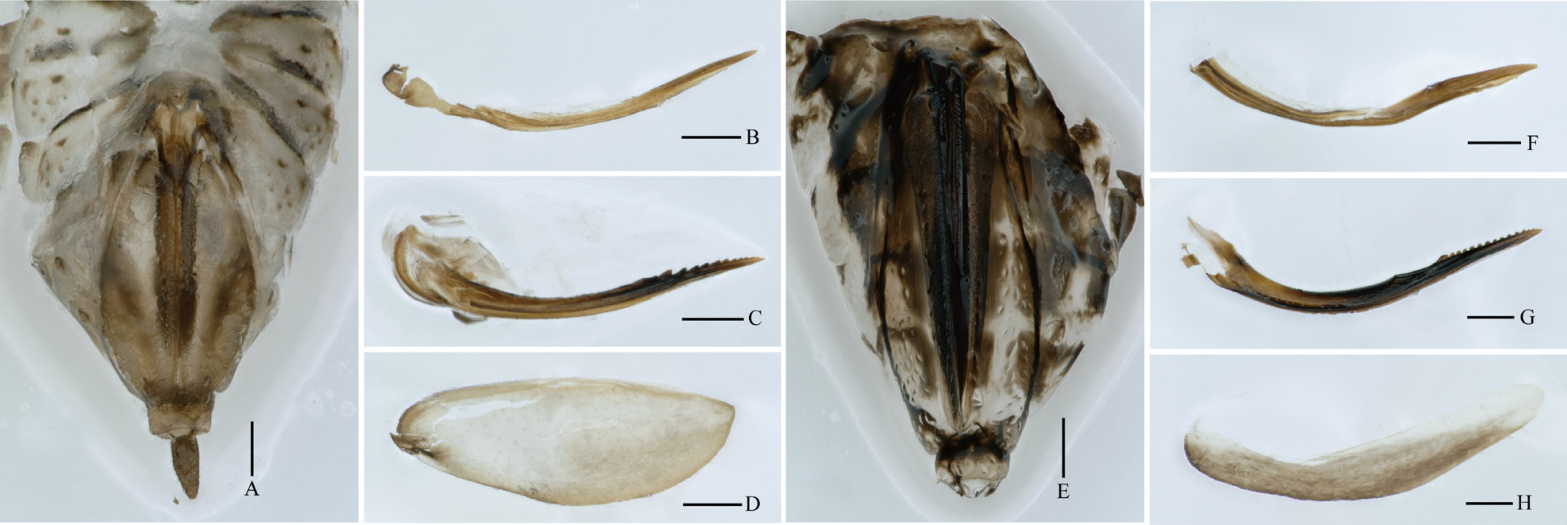
*Stenocranus fodingshanensis* Tang, Li & Chen, sp. nov. **A–D**. Female; **A**. Female genitalia, lateral view; **B**. Gonapophyses VIII, lateral view; **C**. Gonapophyses IX, lateral view; **D**. Gonoplacs, ventral view. *Ceratocranus huaxiensis* Tang, Li & Chen, sp. nov. **E–H**. Female; **E**. Female genitalia, lateral view; **F**. Gonapophyses VIII, lateral view; **G**. Gonapophyses IX, lateral view; **H**. Gonoplacs, ventral view. Scale bars: 0.2 mm**(A–H)**.

**Figure 5.**
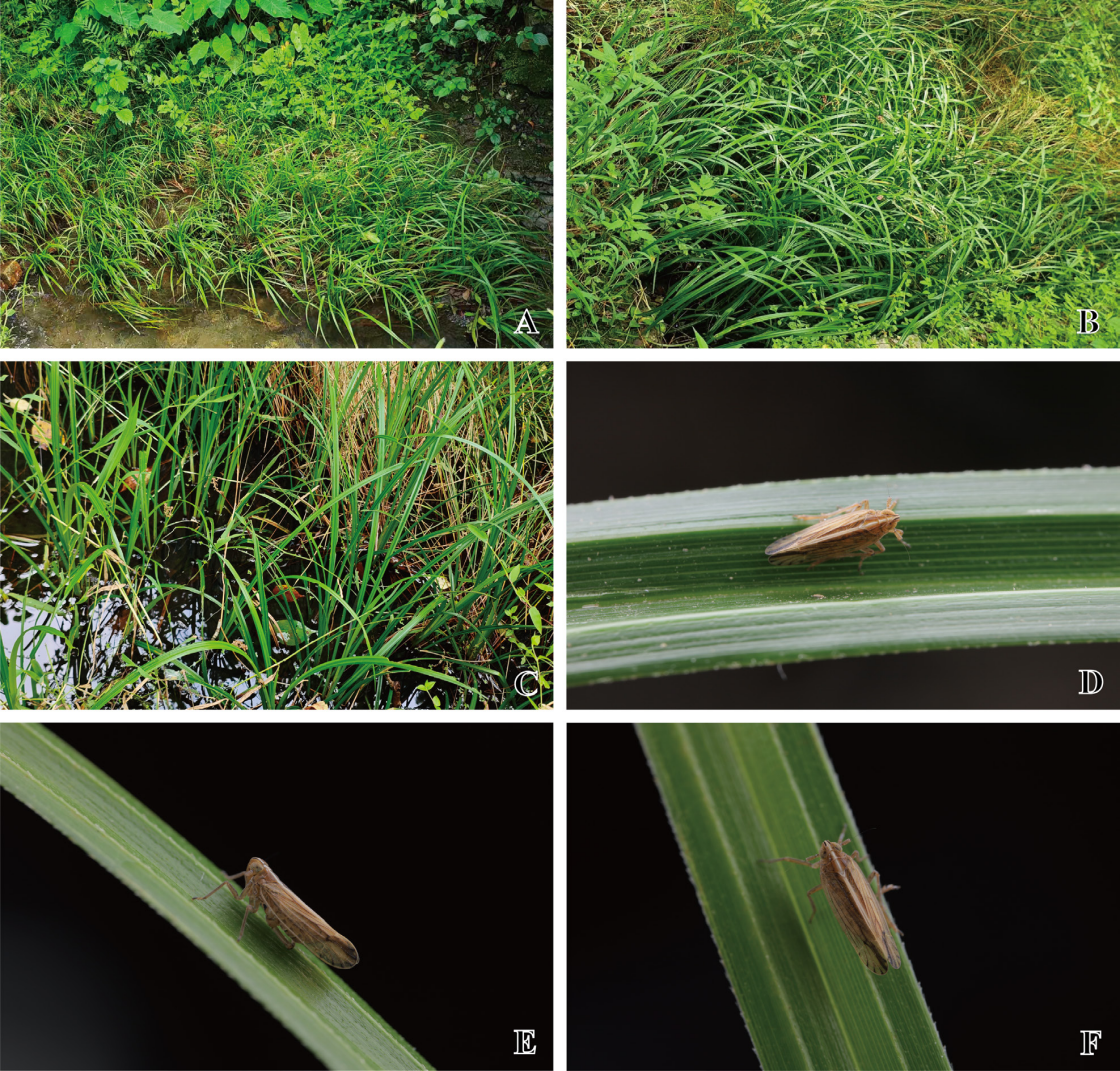
*Stenocranus fodingshanensis* Tang, Li & Chen, sp. nov. **A–C**. Host plants of the type locality; **D–F**. Ecological photos of the adult. Photographs by Ri-Xin Jiang.

#### Diagnosis.

Vertex, pro- and mesonotum with a light-colored medio-longitudinal stripe (Fig. 1A–D); Y-shaped carina indistinct; forewings (Figs 1D, 2A) with T-shaped markings on cross vein near hind margin, and with membrane near hind margins with a fuscous longitudinal stripe, apical veins with dark spots; anal segment (Fig. 2I, J) apical margin curved arch-shaped, lateral margin basal half with hooked spinose process; phallobase (Fig. 2F, G) wide at base, with two curved processes apically, one branch bending at a right angle toward the ventral surface and the other curved dorsad; genital styles (Fig. 2C–E) slender, inner margin prominent at base, and with angular convex near middle; widest in the basal 2/3, upward bifurcation, apical 1/3 toward apex tapering, curved inward to form a diamond-shaped median space.

#### Description.

***Measurements***. Body length including forewing: male 4.0–5.2 mm (*N* = 10), female 4.7–5.8 mm (*N* = 11).

***Coloration***. General color fuscous (Figs 1A, 1B, 5D–F). Eyes fuscous or reddish- brown, vertex, pro- and mesonotum with dirty-white medio-longitudinal stripes, and pro- and mesonotum between median and lateral carinae fuscous. Lateral sides of vertex at apical half, lateral carinae of frons and clypeus with fuscous markings. Median carina of frons brown, bottom and carinae of frons yellowish-white. Forewings (Figs 1D, 2A) hyaline, pale testaceous, veins pale brown, with T-shaped markings on cross vein near hind margin, membrane near hind margin with a fuscous longitudinal stripe, and with dark spots at apical veins. Hindwings (Fig. 2B) concolorous with forewings, wing veins fuscous. Legs testaceous, femora and tibiae with dark brown stripes. Genital styles (Fig. 2C–E) fuscous except base and outer margin black. Aedeagus and phallobase fuscous.

***Head and thorax***. Vertex (Fig. 1A, C) longer in middle line than wide at base (1.45: 1), width at apex narrower than at base (0.86: 1), lateral and median carinae distinct, converge at apex, Y-shaped carina indistinct. Frons (Fig. 1B) longer in middle line than wide at widest portion (2.67: 1), widest at middle of eyes, median carina simple, base of postclypeus wider than apex of frons. Antennae (Fig. 1A, B) with scape wider than long, shorter than pedicel (0.32: 1). Pronotum (Fig. 1A, C) wider than the vertex including the eyes (1.14: 1). Mesonotum midline (Fig. 1A, C) equal in length to combined pronotum and vertex. Forewings (Fig. 2A) extend beyond abdomen, longer than maximal width (3.58: 1), widest in the apical 1/4. Metatibal spur with 20 teeth.

***Male genitalia***. Pygofer ventral margin distinctly longer than dorsal margin in lateral view (Fig. 2I), in posterior view (Fig. 2J) with opening longer than wide, dorsal margin slightly concave medially. Anal segment (Fig. 2I, J) long, apical margin arch-shaped, lateral margin with hooked processes at basal half. Aedeagus with phallobase (Fig. 2F–H), phallus long and slender, curved slightly and tapering towards apices, end of apex round; phallobase wide at base, with two curved processes apically, one branch bending at a right angle toward the ventral surface and the other curved dorsad. Genital styles (Fig. 2C–E) slender, inner margin prominent at base, and with angular convex near middle, widest in the basal 2/3, upward bifurcation, apical 1/3 toward apex tapering, curved inward to form a diamond-shaped space.

***Female genitalia***. Pygofer (Fig. 4A) testaceous; gonapophyses VIII (Fig. 4B) slender in ventral view, weakly serrated on lateroapical parts; gonapophyses IX (Fig. 4C) broad in dorsal view, with 11 acute teeth in apical 1/3; the teeth strongly diverged into asymmetrical two rows; gonoplacs (Fig. 4D) semicircular in ventral view, broad, wider in apical half.

#### Plant association.

*Cyperus* sp. (Cyperaceae) (Fig. 5A–C). All adults were collected from the *Cyperus* sp. near a stream.

#### Distribution.

China (Guizhou Province).

#### Etymology.

This new species is named after the type locality, Fodingshan National Nature Reserve (Guizhou, China); the name is treated as an adjective.

#### Remarks.

This species is similar to *S. chenzhouensis* Ding, 1981, but differs from the latter in: 1) posterior view of genital styles widest in the basal 2/3, apical curved inward (genital styles wide at base, apical curved inward anteriorly, and protrudes dorsal in *S. chenzhouensis*); 2) lateral margin of anal segment with hooked processes at basal half (anal segment lateral margin without processes at basal half in *S. chenzhouensis*); and 3) phallobase with two curved processes apically, one into a right angle directed ventrad, and the other curved dorsally (phallobase with two curved processes in the middle, one into right angle directed ventrad, and the other curved hook-like to the ventral side in *S. chenzhouensis*).

### Ceratocranus

Taxon classification

Animalia

HemipteraDelphacidae

Fujinuma & Hayashi, 2025

B41CACBE-9B0B-5838-9983-F1E82F0CF21B

Ceratocranus Fujinuma & Hayashi, 2025: 330.

#### Type species.

*Stenocranus agamopsyche* Kirkaldy, 1906

#### Diagnosis.

Vertex distinctly projected beyond eyes, about 1.7× as long as wide; anterior margin angulate; lateral margins subparallel; carinae distinctly ridged except median carina obsolete; submedian carinae meeting on fastigium. Rostrum distinctly exceeding mesocoxae. Antennae terete; segment I slightly longer than wide; segment II about 4.3–4.5× as long as wide. Metatibiae with two lateral spines. Hind legs with spinulation: tibial apex with 5 (2+3) spinules, basitarsus with 7 (2+5) spinules, second tarsomere with 5 spinules. Post-tibial spur cultrate, tectiform, shorter than basitarsus, with about 14–20 teeth on posterior margin.

***Male genitalia***: Pygofer rectangular in lateral view, higher than wide of ventral margin, strongly produced caudad in dorsal half of caudal margin; oval in caudal view, higher than wide; diaphragm broad, without armature. Suspensorium rectangular in anterior view, with small protrusion on left dorsal part. Genital styles sinuate in lateral view, forceps-shaped in ventrocaudal view, very broad; basal angles relatively long. Aedeagus very slender, sinuate. Phallobase broad in lateral view, terminating into two downcurved processes; opening for aedeagus on right side. Anal tube rectangular in lateral view; ventral margin bearing one pair of processes.

***Female genitalia***: Pygofer nearly as long as gonoplacs in ventral view, distinctly broader than gonoplacs. Gonocoxae VIII narrow in ventral view, mostly subparallel. Gonapophyses VIII slender in ventral view, nearly smooth on lateroapical parts. Gonapophyses IX relatively broad in dorsal view; dorsal margin with trapezoidal teeth in apical 1/3, small concavity before the teeth. Gonoplacs slender in ventral view, mostly subparallel (Fujinuma and Hayashi 2025).

#### Distribution.

Australian, Oriental and Palearctic regions.

##### Key to Chinese species (males) of the genus *Ceratocranus*

**Table:** 

1	Genital styles inner angle slender, with small processes (Yang 1989: fig. 2I)	***C. agamopsyche* (Kirkaldy, 1906)**
–	Genital styles inner angle broad, without processes (Fig. 3E)	***C. huaxiensis* Tang, Li & Chen, sp. nov**.

### Ceratocranus huaxiensis

Taxon classification

Animalia

HemipteraDelphacidae

Tang, Li & Chen sp. nov.

F125B6F2-A7DE-5652-B54E-F960433E4BBE

https://zoobank.org/83412C6D-C917-45D2-AD9D-B5BB491F1C28

**Figure 6.**
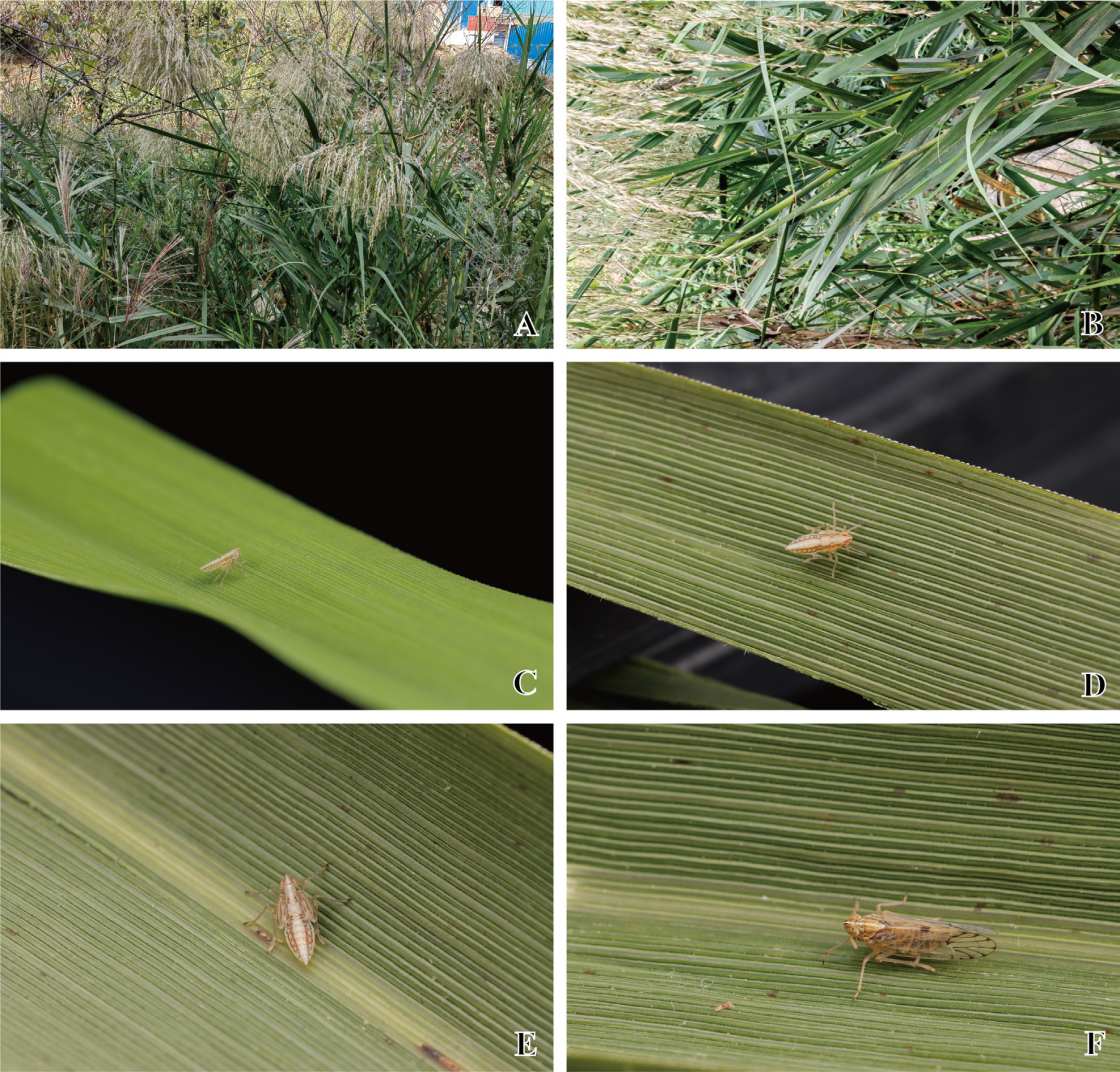
*Ceratocranus huaxiensis* Tang, Li & Chen, sp. nov. **A**, **B**. Host plants of the type locality; **C**. Ecological photos of the third instar nymph; **D**. Ecological photos of the fourth instar nymph; **E**. Ecological photos of the fifth instar nymph; **F**. Ecological photos of the adult. Photographs by Ri-Xin Jiang.

Figs 1E–H, 3, 4E–H, 6C–F

#### Type materials.

***Holotype***: China • ♂: Guizhou Province, Guiyang City, Huaxi District, Jiuan Town; 26°28'N, 106°34'E; sweeping, 17 Oct. 2023; Min Tang, Xiu-Dong Huang and Ri-Xin Jiang leg.; IEGU. ***Paratypes***: China • 14♂♂12♀♀; same data as holotype; IEGU.

#### Diagnosis.

Vertex, pro- and mesonotum (Fig. 1E, G) with a light-colored medio-longitudinal stripe; antennae (Fig. 1E, F) with scape wider than long, shorter than pedicel (0.22: 1); pronotum (Fig. 1H) near lateral margins with small black rounded spots; mesonotum (Fig. 1E, G) equal in length to pronotum and vertex in midline combined; forewings (Figs 1H, 3A) apex of apical veins with fuscous spots; phallobase (Fig. 3D) sheet-like, wide at base, tapering towards apices, skewed to the left, directed ventrad, with a process near middle of ventral margin; genital styles (Fig. 3E) wide at base, with a spinose process at middle part, inner angle broad, without processes, apical half curved inwardly and tapering to apex.

#### Description.

***Measurements***. Body length including forewing: male 5.3–6.1 mm (*N* = 15), female 5.4–6.6 mm (*N* = 12).

***Coloration***. General color testaceous (Figs 1E–H, 6C–F). Ocelli and eyes fuscous. Vertex, pro- and mesonotum with a light-colored medio-longitudinal stripe (Fig. 1E, G). Lateral sides of vertex at apical half, basal genae, frons with black stripes, and clypeus with black markings. Median carinae of frons and clypeus testaceous. Pronotum (Fig. 1E, G, H) between median and lateral carinae with fuscous markings, and with small black rounded spots near lateral margins. Mesonotum between median and lateral carinae testaceous. Forewings (Figs 1H, 3A) hyaline, pale testaceous, ends of apical veins with fuscous markings, wing spots fuscous. Hindwings (Fig. 3B) with forewings concolorous, wing veins pale smoke-brown. Legs yellowish-white, femora and tibiae with dark brown stripes. Anal segment yellowish-brown, stylus fuscous, genital styles black.

***Head and thorax***. Vertex (Fig. 1E, G) longer in middle line than wide at base (1.67: 1), width at apex narrower than at base (0.83: 1), lateral and submedian carinae distinct, converge at apex, Y-shaped carina distinct. Frons (Fig. 1F) longer in middle line than wide at widest portion (2.67: 1), widest at middle of eyes, median carina simple, base of postclypeus slightly wider than apex of frons. Antennae (Fig. 1E, F) with scape wider than long, shorter than pedicel (0.22: 1). Pronotum (Fig. 1E, G) wider than vertex including eyes (1.36: 1). Mesonotum (Fig. 1E, G) equal in length to pronotum and vertex combined in midline. Forewings (Fig. 3A) extend beyond abdomen, longer than maximal width (3.75: 1), widest in the apical 1/4. Metatibal spur with 17–20 teeth.

***Male genitalia***. Pygofer (Fig. 3F) ventral margin distinctly longer than dorsal margin in lateral view, in posterior view (Fig. 3G) with opening longer than wide, dorsal margin slightly concave medially, posterior margin distinctly protruding. Anal segment (Fig. 3F, G) ring-like, with a pair of spinose processes. Aedeagus with phallobase (Fig. 3C, D), phallus long and slender, curved slightly and tapering towards apices, apical portion very weak; phallobase sheet-like, wide at base, tapering towards apices, skewed to the left, directed ventrad, with a process near middle of ventral margin. Genital styles (Fig. 3E) wide at base, with a spinose process medially, apical half inwardly curved and tapering to apex.

***Female genitalia***. Pygofer (Fig. 4E) fuscous; gonapophyses VIII (Fig. 4F) narrow in ventral view, mostly subparallel, slender in ventral view, weakly serrated on lateroapical parts; gonapophyses IX (Fig. 4G) relatively broad in dorsal view; with 15 acute teeth in apical 1/3, small concavity before the teeth; gonoplacs (Fig. 4H) slender in ventral view, mostly subparallel.

#### Plant association.

*Phragmites australis* (Cav.) Trin. ex Steud. (Poaceae) (Fig. 6A, B). All adults were collected from the *P. australis* along a river.

#### Distribution.

China (Guizhou Province).

#### Etymology.

This new species is named after the type locality, Huaxi District (Guiyang City, Guizhou, China); the name is treated as an adjective.

#### Remarks.

This species is similar to *C. agamopsyche* (Kirkaldy, 1906), but differs from the latter in: 1) mesonotum equal in length to pronotum and vertex combined in midline (mesonotum longer than pronotum and vertex combined in midline in *C. agamopsyche*); 2) genital styles inner angle broad, without process (genital styles inner angle slender, inner margin at base with a process in *C. agamopsyche*); and 3) phallobase with a straight process near middle of ventral margin (phallobase with a curved process near middle of ventral margin in *C. agamopsyche*).

## Discussion

All species of Delphacidae are phytophagous, sucking fluids from leaves, stems, or roots. They primarily feed on monocots, but also include dicots and even mosses, with a particular preference for grasses and sedge plants (Wilson et al. 1994). Among which Tropidocephalini have been reported to be important or potential pests of bamboo (Ding 2006; Hou and Chen 2010, 2013; Li et al. 2010; Yang and Chen 2011; Li et al. 2018, 2023; Chen and Yang 2023; Chen et al. 2024). At present, there are relatively few reports on the host plants of the Stenocranine delphacids. Most records indicate that these hosts are primarily grasses (Poaceae) and sedges (Cyperaceae), with two ferns and *Equisetum* L., 1887 (Equisetaceae) also noted. North American stenocranines are predominantly associated with *Carex* L., 1753 (Cyperaceae) and *Arundinaria* Michx., 1803 (Poaceae: Bambusoideae). In contrast, Old World stenocranines are mainly reported from *Carex*, grasses (Poaceae), and *Phragmites* (Bartlett 2009). Fujinuma and Hayashi (2025) reported that the host plants of Japanese stenocranine delphacids are primarily species from the genus *Phragmites* and *Cyperus*. Within the genus *Stenocranus*, *S. arundineus* Metcalf, 1923 and *S. similis* Crawford, 1914 feed on grasses from the genus *Arundinaria*, with *S. similis* Crawford, 1914 also found on *Eleocharis quadrangulata* (Michaux) Roem. & Schult., 1817. Additionally, *S. lautus* Van Duzee, 1897, *S. brunneus* Beamer, 1946, and *S. unipunctatus* have been reported to feed on *Carex*, while *S. lautus* Van Duzee, 1897 also develops on *Cyperus* (Bartlett and Wheeler 2007).

In this paper, we describe two new species from China and provisionally place them in the genera *Stenocranus* and *Ceratocranus*. Based on data from published information and our field surveys, the species of stenocranine delphacids of China were known predominantly from the Oriental and Palaearctic regions, but most species are known only from their type locality (Ding 2006); the actual distribution range of most species is still unclear. Therefore, further collection and investigation remain necessary to enrich their distribution range, and many more taxa remain to be discovered and described to fill faunistic gaps.

## Supplementary Material

XML Treatment for Stenocranus

XML Treatment for Stenocranus fodingshanensis

XML Treatment for Ceratocranus

XML Treatment for Ceratocranus huaxiensis

## Additional information

### Artificial Intelligence (AI) use

The authors accept full responsibility for the content of the manuscript, including the disclosure of any use of AI.

No AI tools were used in the preparation of this manuscript.

### Author ORCIDs

M. Tang https://orcid.org/0009-0006-7371-9843

N. Gong https://orcid.org/0000-0002-8878-5337

Z-Z. Li https://orcid.org/0000-0003-1609-9992

L. Yang https://orcid.org/0000-0002-7841-5156

S.-S. Lv https://orcid.org/0000-0001-5353-5082

J.-K. Long https://orcid.org/0000-0002-4576-9919

Z.-M. Chang https://orcid.org/0000-0002-7247-9706

X.-S. Chen https://orcid.org/0000-0001-9801-0343

### Data availability

All of the data that support the findings of this study are available in the main text.
